# Copy Right in Lectures

**Published:** 1842-03

**Authors:** 


*Copy Right in Lectures.*—The question as to what right a lecturer has in his oral lectures—and whether he can prevent their being published by any one who may attend them and take notes for the purpose, has recently been brought before the courts of our city. Dr. Mott applied to the court of chancery for an injunction restraining Dr. James A. Houston from publishing his, Dr. Mott's Lectures, in the Lancet. This injunction was sought on two grounds, first, that Dr. Mott had hired Dr. H. to take notes of the lectures, and paid him a valuable consideration therefor, and that part of the bargain was, that no public use should be made of the notes. The second ground was the general principle, that a public lecturer has a right in his lectures, and that it is a violation of that right for any person to publish the lecture, or notes of it; that notes can only be taken for the individual benefit of the student, and not to be published for gain. These were the grounds on which Dr. Mott sought to prevent the publication of his lectures in the Lancet. In support of the application, David Graham, Esq., appeared before the assistant Vice-Chancellor, Murray Hoffman, Esq., and Dr. Houston was defended by Mr. Sherwood. The case was fully argued on both sides, and on Wednesday last the opinion of the court was given. The Assistant Vice-Chancellor held, that the State Courts had no jurisdiction in the matter, it belonging exclusively to the U. S. Courts; upon that ground the injunction was, substantially, dissolved. The learned judge however expressed *a very decided opinion, that Dr. Mott could restrain the Editor of the Lancet from publishing his lectures*, on the ground of the bargain alleged by Dr. M. to have been made, whereby Dr. H. expressly stipulated that he would make no such use of the notes, which Dr. M. employed him to take. As to the general question, whether a public lecturer can restrain a student or other person from publishing notes he may take of the lecture, the Court had great doubts. The decision of Lord Eldon in the case of Mr. Abernethy, versus the Lancet,* could not be considered as settling the law upon this matter, although it is quoted with approbation by Judge Story and Chancellor Kent.†—*N. Y. Med. Gaz.*

---

* It seems to be a very general opinion among medical men, that in this case the English Court of Chancery decided against the right of a lecturer to prevent the republication of his lectures; this is a mistake, the decision was exactly the reverse. Lord Eldon gave a long and very able opinion in favor of Mr. Abernethy, in which he sustained fully the ground taken by the plaintiff, viz.; "that a lecturer had a right in his lectures, which none of his hearers could invade, by publishing notes of his lectures." At a subsequent stage of the proceedings the contest was abandoned by Mr. Abernethy, and consequently the injunction was dissolved by default, no one appearing to support it. As the law now stands in England, the right in question is possessed by every public lecturer; the expediency of exercising this right is of course another and a very different question.

† We understand that Dr. M. has appealed from this decision, and proposes to pursue the matter in the higher courts.

